# (*E*)-1-Methyl-4-[2-(1-naphth­yl)vin­yl]pyridinium 4-chloro­benzene­sulfonate^1^

**DOI:** 10.1107/S1600536809047734

**Published:** 2009-11-18

**Authors:** Suchada Chantrapromma, Kullapa Chanawanno, Hoong-Kun Fun

**Affiliations:** a Crystal Materials Research Unit, Department of Chemistry, Faculty of Science, Prince of Songkla University, Hat-Yai, Songkhla 90112, Thailand; b X-ray Crystallography Unit, School of Physics, Universiti Sains Malaysia, 11800 USM, Penang, Malaysia

## Abstract

In the title compound, C_18_H_16_N^+^·C_6_H_4_ClO_3_S^−^, the cation exists in an *E* configuration with respect to the central C=C bond. The naphthalene ring system is slightly bent, the dihedral angle between the two aromatic rings being 3.71 (14)°. The whole cation is twisted, the dihedral angles between the pyridinium and the two aromatic rings of the naphthalene ring system being 47.44 (14) and 50.81 (14)°. The pyridinium ring and the benzene ring of the anion are inclined to each other at a dihedral angle of 68.21 (13)°. In the crystal structure, the cations and anions are arranged alternately with the cations stacked in an anti-parallel manner along the *c* axis and the anions linked into chains along the same direction. The cations are linked to the anions by weak C—H⋯O inter­actions, forming a three-dimensional network. The crystal structure is further stabilized by C—H⋯π inter­actions and π–π contacts with centroid–centroid distances of 3.6374 (16) and 3.6733 (17) Å. A short Cl⋯O contact [3.108 (2) Å] is also present.

## Related literature

For bond-length data, see: Allen *et al.* (1987 ▶). For background to NLO materials, see: Amila *et al.* (2004 ▶); Babu *et al.* (2009 ▶); Chandramohan *et al.* (2008 ▶); Martin *et al.* (2002 ▶); Srinivasan *et al.* (2007 ▶); Yildiz *et al.* (2009 ▶). For related structures, see: Chantrapromma *et al.* (2007 ▶, 2009 ▶); Fun *et al.* (2009 ▶). For the stability of the temperature controller used in the data collection, see: Cosier & Glazer (1986 ▶).
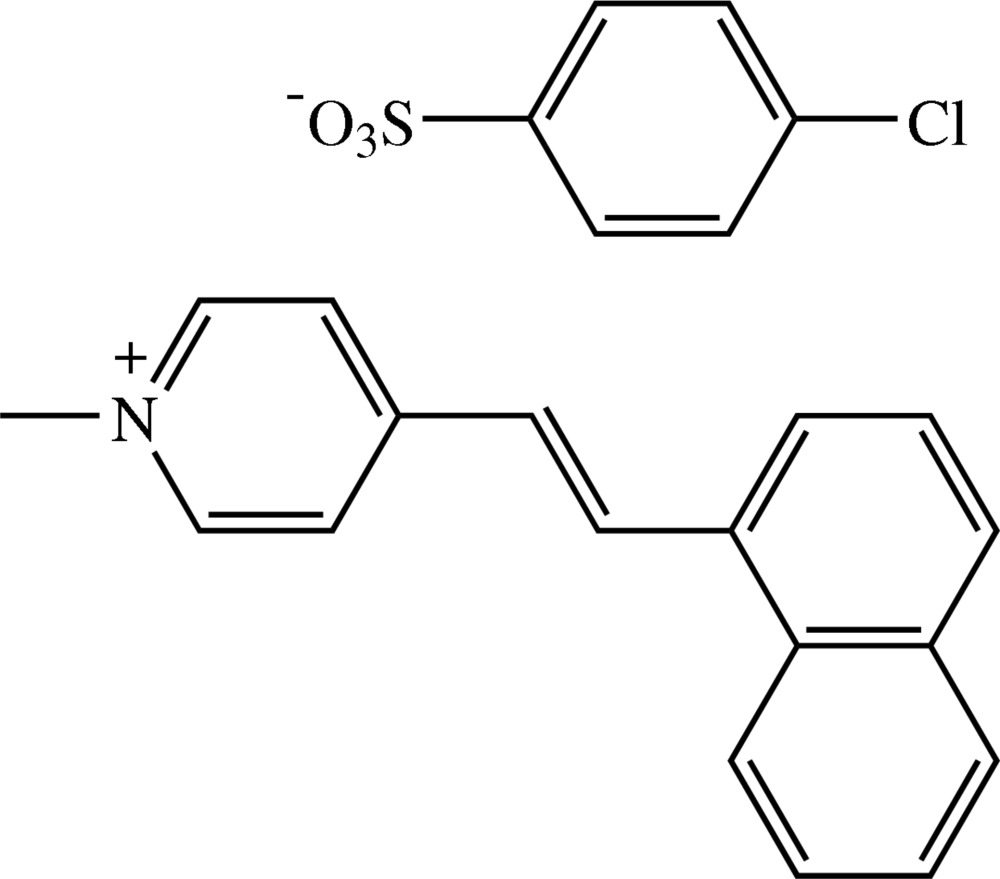


## Experimental

### 

#### Crystal data

C_18_H_16_N^+^·C_6_H_4_ClO_3_S^−^
*M* *_r_* = 437.93
Orthorhombic, 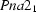

*a* = 12.3379 (8) Å
*b* = 21.8466 (16) Å
*c* = 7.5032 (5) Å
*V* = 2022.4 (2) Å^3^
*Z* = 4
Mo *K*α radiation
μ = 0.32 mm^−1^
*T* = 100 K
0.52 × 0.15 × 0.03 mm

#### Data collection

Bruker APEXII CCD area-detector diffractometer
Absorption correction: multi-scan (**SADABS**; Bruker, 2005 ▶) *T*_min_ = 0.852, *T*_max_ = 0.990
26247 measured reflections
5881 independent reflections
5018 reflections with *I* > 2σ(*I*)
*R*_int_ = 0.045

#### Refinement

*R*[*F* ^2^ > 2σ(*F* ^2^)] = 0.050
*wR*(*F* ^2^) = 0.119
*S* = 1.03
5881 reflections
272 parameters
1 restraint
H-atom parameters constrained
Δρ_max_ = 0.79 e Å^−3^
Δρ_min_ = −0.32 e Å^−3^
Absolute structure: Flack (1983 ▶), 2716 Friedel pairs
Flack parameter: 0.01 (6)

### 

Data collection: *APEX2* (Bruker, 2005 ▶); cell refinement: *SAINT* (Bruker, 2005 ▶); data reduction: *SAINT*; program(s) used to solve structure: *SHELXTL* (Sheldrick, 2008 ▶); program(s) used to refine structure: *SHELXTL*; molecular graphics: *SHELXTL*; software used to prepare material for publication: *SHELXTL* and *PLATON* (Spek, 2009 ▶).

## Supplementary Material

Crystal structure: contains datablocks global, I. DOI: 10.1107/S1600536809047734/is2479sup1.cif

Structure factors: contains datablocks I. DOI: 10.1107/S1600536809047734/is2479Isup2.hkl

Additional supplementary materials:  crystallographic information; 3D view; checkCIF report

## Figures and Tables

**Table 1:** Hydrogen-bond geometry (Å, °)

*D*—H⋯*A*	*D*—H	H⋯*A*	*D*⋯*A*	*D*—H⋯*A*
C5—H5*A*⋯O3	0.93	2.51	3.370 (3)	153
C11—H11*A*⋯O1^i^	0.93	2.34	3.267 (3)	178
C14—H14*A*⋯O1^i^	0.93	2.38	3.260 (3)	158
C15—H15*A*⋯O2^ii^	0.93	2.42	3.285 (3)	155
C17—H17*A*⋯O3^iii^	0.93	2.43	3.348 (3)	171
C18—H18*A*⋯O2^ii^	0.96	2.45	3.372 (4)	160
C20—H20*A*⋯O1^iv^	0.93	2.34	3.226 (4)	159
C22—H22*A*⋯O2^v^	0.93	2.52	3.277 (3)	139
C1—H1*A*⋯*Cg*4^iii^	0.93	2.98	3.682 (3)	133
C3—H3*A*⋯*Cg*4^vi^	0.93	2.87	3.651 (3)	142
C6—H6*A*⋯*Cg*3^vii^	0.93	2.82	3.593 (3)	141

Symmetry codes: (i) 
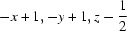
; (ii) 
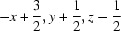
; (iii) 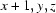; (iv) 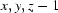; (v) 
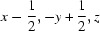
; (vi) 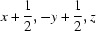; (vii) 
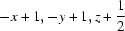
. *Cg*1, *Cg*2, *Cg*3 and *Cg*4 are the centroids of the N1/C13–C17, C1–C4/C9/C10, C4–C9 and C19–C24 rings, respectively.

**Fig. 1.**
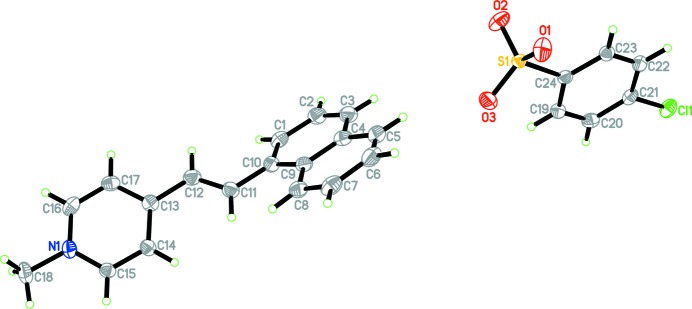
The molecular structure of the title compound, with 50% probability displacement ellipsoids and the atom-numbering scheme.

**Fig. 2.**
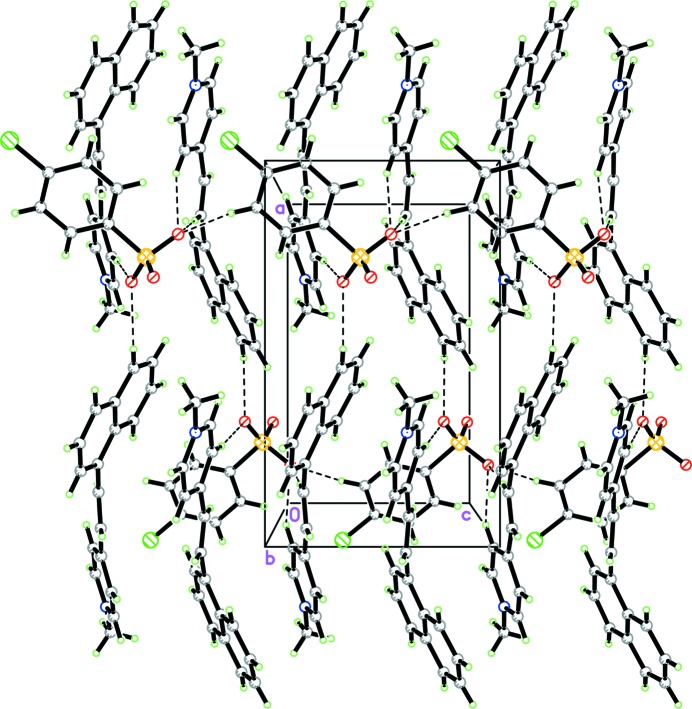
The crystal packing of the title compound viewed down the b axis. Weak C—H···O interactions are shown as dashed lines.

## supplementary crystallographic information

### Comment

A variety of compounds have been investigated for their nonlinear optical (NLO) properties due to the important applications of such materials in the domain of opto-electronics and photonics (Amila *et al.*, 2004; Chandramohan *et al.*, 2008; Martin *et al.*, 2002; Srinivasan *et al.*, 2007). During the course of our NLO materials research, we found that styryl and napthalenyl are suitable units to be applied for NLO compounds and we have previously synthesized and reported the crystal structure of the NLO pyridinium salt containing these two units *i.e.* (*E*)-1-methyl-4-(2-(naphthalen-1-yl)vinyl)pyridinium 4-bromobenzenesulfonate [compound (I); Chantrapromma *et al.*, 2009]. With the aim to investigate the influence of the anion counter part to NLO properties, the title compound (II) was synthesized by changing the 4-bromobenzenesulfonate anionic counter part in compound (I) to the 4-chlorobenzenesulfonate. The title compound (II) crystallizes in the orthorhombic non-centrosymmetric space group *P*na2_1_ [same as compound (I)] therefore it should exhibit second-order nonlinear optical properties according to the literature knowledge (Babu *et al.*, 2009; Yildiz *et al.*, 2009).

Figure 1 shows the asymmetric unit of (II) which consists of a C_18_H_16_N^+^ cation and a C_6_H_4_ClO_3_S^-^ anion. The cation exists in an *E* configuration with respect to the C11═C12 double bond [1.341 (4) Å] and the torsion angle C10–C11–C12–C13 is 179.6 (3)°. The naphthalenyl moiety is slightly bent which can be reflected by the dihedral angle between the two aromatic C1–C4/C9–C10 and C4–C9 rings being 3.68 (14)° [the corresponding value is 5.0 (5)° for major component and 5.7 (10)° for minor component in compound (I) which is a disordered structure; Chantrapromma *et al.*, 2009]. The whole molecule of cation is twisted with dihedral angles between the pyridinium and the two aromatic C1–C4/C9–C10 and C4–C9 rings being 47.44 (14) and 50.81 (14)°, respectively [56.3 (5) and 51.4 (5)° for major component and 52.2 (11) and 53.4 (11)° for minor component in compound (I); Chantrapromma *et al.*, 2009]. The orientation of the ethenyl unit can be described as atom C11 lies on the same plane with naphthalenyl moiety with the *rms* deviation of 0.028 (3) Å whereas atom C12 lies on the same plane with the pyridinium ring with the *rms* deviation of 0.017 (3) Å and the torsion angles C8–C9–C10–C11 of -0.5 (4)° and C11–C12–C13–C17 of -11.4 (5)°. The cation and anion are inclined to each other with a dihedral angle of 68.21 (13)° between the pyridinium and C19–C24 rings [the corresponding value is 85.0 (4)° for major component and 71.5 (9)° for minor component in compound (I); Chantrapromma *et al.*, 2009]. The bond lengths in (II) are in normal ranges (Allen *et al.*, 1987) and comparable to the closely related structures (Chantrapromma *et al.*, 2009; Fun *et al.*, 2009).

In the crystal packing (Fig. 2), all O atoms of the sulfonate group are involved in weak C—H···O interactions (Table 1). The cations and anions are alternately arranged with the cations stacked in an antiparallel manner along the *c* axis and the anions linked together into chains along the same direction. The cations are linked to the anions by weak C—H···O interactions (Table 1) forming a 3D network. The crystal structure is further stabilized by C—H···π interactions (Table 1). π···π interactions with the distances Cg_1_···Cg_2_ = 3.6733 (17) Å and Cg_1_···Cg_3_ = 3.6374 (16) Å are also observed (symmetry code for both Cg···Cg interactions: 2-x, 1-y,-1/2+z); Cg_1_, Cg_2_, Cg_3_ and Cg_4_ are the centroids of the N1/C13–C17, C1–C4/C9–C10, C4–C9 and C19–C24 rings, respectively. A short Cl1···O2 [3.108 (2) Å] contact is also present.

### Experimental

(*E*)-1-Methyl-4-(2-(naphthalen-1-yl)vinyl)pyridinium iodide (compound A)(0.22 g, 0.58 mmol) which was prepared according to the previous method (Fun *et al.*, 2009) was mixed with silver 4-chlorobenzenesulfonate (Chantrapromma *et al.*, 2007) (0.20 g, 0.58 mmol) in methanol (100 ml) and stirred for 0.5 h. The precipitate of silver iodide which formed was filtered and the filtrate was evaporated to give the title compound as a yellow solid. Yellow needle-shaped single crystals of the title compound suitable for *x*-ray structure determination were recrystallized from methanol by slow evaporation at room temperature over a few weeks (m.p. 476-477 K).

### Refinement

All H atoms were positioned geometrically and allowed to ride on their parent atoms, with d(C-H) = 0.93 Å for aromatic and CH and 0.96 Å for CH_3_ atoms. The *U*_iso_(H) values were constrained to be 1.5*U*_eq_ of the carrier atom for methyl H atoms and 1.2*U*_eq_ for the remaining H atoms. A rotating group model was used for the methyl groups. The highest residual electron density peak is located at 0.80 Å from S1 and the deepest hole is located at 0.66 Å from C11.

### Crystal data

**Table:**

C_18_H_16_N^+^·C_6_H_4_ClO_3_S^−^	*D*_x_ = 1.438 Mg m^−^^3^
*M**_r_* = 437.93	Melting point = 476–477 K
Orthorhombic, *P**n**a*2_1_	Mo *K*α radiation, λ = 0.71073 Å
Hall symbol: P 2c -2n	Cell parameters from 5881 reflections
*a* = 12.3379 (8) Å	θ = 2.5–30.0°
*b* = 21.8466 (16) Å	µ = 0.32 mm^−^^1^
*c* = 7.5032 (5) Å	*T* = 100 K
*V* = 2022.4 (2) Å^3^	Needle, yellow
*Z* = 4	0.52 × 0.15 × 0.03 mm
*F*(000) = 912	

### Data collection

**Table:**

Bruker APEXII CCD area-detector diffractometer	5881 independent reflections
Radiation source: sealed tube	5018 reflections with *I* > 2σ(*I*)
graphite	*R*_int_ = 0.045
φ and ω scans	θ_max_ = 30.0°, θ_min_ = 2.5°
Absorption correction: multi-scan (*SADABS*; Bruker, 2005)	*h* = −17→17
*T*_min_ = 0.852, *T*_max_ = 0.990	*k* = −25→30
26247 measured reflections	*l* = −10→10

### Refinement

**Table:**

Refinement on *F*^2^	Secondary atom site location: difference Fourier map
Least-squares matrix: full	Hydrogen site location: inferred from neighbouring sites
*R*[*F*^2^ > 2σ(*F*^2^)] = 0.050	H-atom parameters constrained
*wR*(*F*^2^) = 0.119	*w* = 1/[σ^2^(*F*_o_^2^) + (0.0563*P*)^2^ + 1.1415*P*] where *P* = (*F*_o_^2^ + 2*F*_c_^2^)/3
*S* = 1.03	(Δ/σ)_max_ = 0.001
5881 reflections	Δρ_max_ = 0.79 e Å^−^^3^
272 parameters	Δρ_min_ = −0.32 e Å^−^^3^
1 restraint	Absolute structure: Flack (1983), 2716 Friedel pairs
Primary atom site location: structure-invariant direct methods	Flack parameter: 0.01 (6)

### Special details

**Table:**

Experimental. The crystal was placed in the cold stream of an Oxford Cryosystems Cobra open-flow nitrogen cryostat (Cosier & Glazer, 1986) operating at 100.0 (1) K.
Geometry. All esds (except the esd in the dihedral angle between two l.s. planes) are estimated using the full covariance matrix. The cell esds are taken into account individually in the estimation of esds in distances, angles and torsion angles; correlations between esds in cell parameters are only used when they are defined by crystal symmetry. An approximate (isotropic) treatment of cell esds is used for estimating esds involving l.s. planes.
Refinement. Refinement of F^2^ against ALL reflections. The weighted R-factor wR and goodness of fit S are based on F^2^, conventional R-factors R are based on F, with F set to zero for negative F^2^. The threshold expression of F^2^ > 2sigma(F^2^) is used only for calculating R-factors(gt) etc. and is not relevant to the choice of reflections for refinement. R-factors based on F^2^ are statistically about twice as large as those based on F, and R- factors based on ALL data will be even larger.

### Fractional atomic coordinates and isotropic or equivalent isotropic displacement parameters (Å^2^)

**Table:**

	*x*	*y*	*z*	*U*_iso_*/*U*_eq_	
Cl1	−0.09030 (5)	0.23662 (3)	0.30957 (10)	0.02946 (15)	
S1	0.22593 (4)	0.33346 (2)	0.90465 (9)	0.01933 (12)	
O1	0.15823 (14)	0.35562 (10)	1.0481 (3)	0.0330 (5)	
O2	0.28543 (16)	0.27849 (8)	0.9519 (3)	0.0334 (5)	
O3	0.29257 (15)	0.37986 (9)	0.8218 (3)	0.0345 (5)	
N1	1.27331 (16)	0.61843 (10)	0.6247 (3)	0.0254 (5)	
C1	0.8387 (2)	0.39813 (12)	0.5753 (4)	0.0285 (6)	
H1A	0.9051	0.3871	0.5262	0.034*	
C2	0.7561 (2)	0.35470 (13)	0.5858 (4)	0.0329 (6)	
H2A	0.7677	0.3153	0.5428	0.040*	
C3	0.6593 (2)	0.36949 (13)	0.6579 (4)	0.0329 (6)	
H3A	0.6043	0.3404	0.6621	0.040*	
C4	0.6405 (2)	0.42940 (13)	0.7278 (4)	0.0285 (6)	
C5	0.5414 (2)	0.44348 (13)	0.8145 (4)	0.0331 (6)	
H5A	0.4872	0.4140	0.8209	0.040*	
C6	0.5245 (2)	0.49983 (14)	0.8888 (4)	0.0346 (6)	
H6A	0.4606	0.5079	0.9502	0.042*	
C7	0.6038 (2)	0.54547 (13)	0.8719 (4)	0.0347 (7)	
H7A	0.5909	0.5842	0.9191	0.042*	
C8	0.7004 (2)	0.53381 (12)	0.7868 (4)	0.0307 (6)	
H8A	0.7517	0.5647	0.7753	0.037*	
C9	0.7219 (2)	0.47452 (12)	0.7161 (4)	0.0254 (5)	
C10	0.8240 (2)	0.45755 (12)	0.6368 (4)	0.0270 (6)	
C11	0.9108 (2)	0.50246 (12)	0.6253 (4)	0.0269 (6)	
H11A	0.8919	0.5431	0.6063	0.032*	
C12	1.0164 (2)	0.48887 (12)	0.6404 (4)	0.0287 (6)	
H12A	1.0349	0.4482	0.6602	0.034*	
C13	1.1056 (2)	0.53391 (12)	0.6279 (4)	0.0246 (5)	
C14	1.09004 (19)	0.59384 (11)	0.5685 (4)	0.0237 (5)	
H14A	1.0217	0.6060	0.5300	0.028*	
C15	1.17418 (19)	0.63513 (11)	0.5659 (4)	0.0226 (5)	
H15A	1.1628	0.6746	0.5236	0.027*	
C16	1.2913 (2)	0.56030 (14)	0.6814 (4)	0.0295 (6)	
H16A	1.3602	0.5492	0.7195	0.035*	
C17	1.2101 (2)	0.51791 (13)	0.6835 (4)	0.0262 (5)	
H17A	1.2243	0.4782	0.7220	0.031*	
C18	1.3610 (2)	0.66379 (14)	0.6319 (5)	0.0351 (7)	
H18A	1.3364	0.7016	0.5810	0.053*	
H18B	1.4222	0.6490	0.5657	0.053*	
H18C	1.3817	0.6704	0.7537	0.053*	
C19	0.15667 (19)	0.31883 (11)	0.5567 (4)	0.0236 (5)	
H19A	0.2191	0.3400	0.5250	0.028*	
C20	0.0877 (2)	0.29687 (11)	0.4251 (4)	0.0252 (5)	
H20A	0.1034	0.3034	0.3053	0.030*	
C21	−0.0051 (2)	0.26499 (11)	0.4749 (4)	0.0243 (5)	
C22	−0.03135 (19)	0.25634 (12)	0.6525 (4)	0.0243 (5)	
H22A	−0.0946	0.2358	0.6839	0.029*	
C23	0.03806 (19)	0.27873 (11)	0.7836 (4)	0.0218 (5)	
H23A	0.0212	0.2733	0.9034	0.026*	
C24	0.13284 (19)	0.30930 (11)	0.7354 (3)	0.0200 (5)	

### Atomic displacement parameters (Å^2^)

**Table:**

	*U*^11^	*U*^22^	*U*^33^	*U*^12^	*U*^13^	*U*^23^
Cl1	0.0291 (3)	0.0304 (3)	0.0289 (3)	0.0041 (2)	−0.0085 (3)	−0.0063 (3)
S1	0.0201 (2)	0.0166 (2)	0.0212 (3)	0.0019 (2)	0.0004 (2)	0.0021 (2)
O1	0.0269 (9)	0.0453 (11)	0.0269 (11)	0.0013 (8)	−0.0008 (8)	−0.0109 (10)
O2	0.0385 (11)	0.0259 (9)	0.0359 (12)	0.0103 (8)	−0.0144 (9)	−0.0033 (8)
O3	0.0330 (9)	0.0374 (11)	0.0330 (11)	−0.0125 (8)	−0.0062 (9)	0.0090 (10)
N1	0.0193 (9)	0.0272 (11)	0.0296 (12)	−0.0021 (8)	0.0001 (9)	−0.0025 (9)
C1	0.0293 (12)	0.0293 (13)	0.0268 (14)	0.0079 (10)	−0.0029 (11)	−0.0006 (12)
C2	0.0407 (15)	0.0218 (12)	0.0362 (17)	−0.0005 (11)	−0.0091 (13)	−0.0015 (12)
C3	0.0385 (15)	0.0263 (13)	0.0340 (17)	−0.0031 (11)	−0.0076 (13)	0.0042 (12)
C4	0.0308 (12)	0.0292 (13)	0.0256 (14)	0.0020 (11)	−0.0024 (11)	0.0058 (11)
C5	0.0294 (12)	0.0393 (15)	0.0305 (15)	−0.0015 (11)	−0.0047 (13)	0.0090 (14)
C6	0.0268 (12)	0.0488 (16)	0.0283 (15)	0.0093 (12)	0.0010 (13)	0.0068 (14)
C7	0.0346 (13)	0.0322 (14)	0.0374 (17)	0.0130 (11)	−0.0045 (12)	−0.0006 (13)
C8	0.0294 (12)	0.0215 (12)	0.0413 (17)	0.0039 (10)	−0.0046 (12)	0.0033 (12)
C9	0.0264 (12)	0.0246 (12)	0.0252 (14)	0.0019 (10)	0.0000 (11)	0.0050 (11)
C10	0.0265 (12)	0.0220 (12)	0.0325 (15)	−0.0011 (10)	−0.0055 (11)	0.0016 (11)
C11	0.0285 (12)	0.0227 (12)	0.0297 (15)	−0.0004 (10)	0.0006 (11)	0.0013 (11)
C12	0.0322 (13)	0.0228 (13)	0.0313 (16)	0.0018 (10)	−0.0036 (12)	0.0014 (11)
C13	0.0248 (11)	0.0215 (11)	0.0274 (14)	0.0027 (9)	0.0025 (11)	−0.0030 (10)
C14	0.0203 (10)	0.0222 (11)	0.0285 (14)	0.0013 (9)	0.0043 (10)	−0.0010 (11)
C15	0.0226 (11)	0.0215 (11)	0.0239 (13)	0.0011 (9)	0.0037 (10)	0.0019 (10)
C16	0.0255 (12)	0.0361 (15)	0.0268 (14)	0.0087 (11)	−0.0039 (11)	−0.0023 (12)
C17	0.0303 (13)	0.0250 (12)	0.0233 (13)	0.0046 (10)	0.0035 (11)	0.0021 (10)
C18	0.0219 (12)	0.0359 (15)	0.0475 (19)	−0.0072 (11)	−0.0005 (12)	−0.0064 (14)
C19	0.0233 (11)	0.0217 (11)	0.0256 (13)	−0.0005 (9)	−0.0004 (10)	0.0017 (10)
C20	0.0305 (12)	0.0242 (12)	0.0210 (13)	0.0039 (9)	0.0011 (11)	0.0013 (11)
C21	0.0243 (11)	0.0210 (11)	0.0277 (14)	0.0038 (9)	−0.0066 (10)	−0.0020 (10)
C22	0.0204 (11)	0.0245 (12)	0.0280 (14)	0.0005 (9)	−0.0020 (10)	0.0013 (11)
C23	0.0215 (10)	0.0212 (11)	0.0226 (14)	0.0026 (9)	0.0005 (10)	0.0003 (10)
C24	0.0206 (10)	0.0182 (11)	0.0211 (12)	0.0024 (9)	−0.0007 (9)	0.0021 (9)

### Geometric parameters (Å, °)

**Table:**

Cl1—C21	1.740 (3)	C10—C11	1.455 (4)
S1—O1	1.446 (2)	C11—C12	1.341 (4)
S1—O3	1.446 (2)	C11—H11A	0.9300
S1—O2	1.4514 (18)	C12—C13	1.480 (4)
S1—C24	1.792 (3)	C12—H12A	0.9300
N1—C15	1.350 (3)	C13—C14	1.396 (4)
N1—C16	1.358 (4)	C13—C17	1.400 (4)
N1—C18	1.468 (3)	C14—C15	1.375 (3)
C1—C10	1.389 (4)	C14—H14A	0.9300
C1—C2	1.395 (4)	C15—H15A	0.9300
C1—H1A	0.9300	C16—C17	1.364 (4)
C2—C3	1.351 (4)	C16—H16A	0.9300
C2—H2A	0.9300	C17—H17A	0.9300
C3—C4	1.429 (4)	C18—H18A	0.9600
C3—H3A	0.9300	C18—H18B	0.9600
C4—C9	1.410 (4)	C18—H18C	0.9600
C4—C5	1.419 (4)	C19—C24	1.388 (4)
C5—C6	1.367 (4)	C19—C20	1.389 (4)
C5—H5A	0.9300	C19—H19A	0.9300
C6—C7	1.402 (4)	C20—C21	1.392 (4)
C6—H6A	0.9300	C20—H20A	0.9300
C7—C8	1.376 (4)	C21—C22	1.384 (4)
C7—H7A	0.9300	C22—C23	1.393 (4)
C8—C9	1.425 (4)	C22—H22A	0.9300
C8—H8A	0.9300	C23—C24	1.395 (3)
C9—C10	1.441 (4)	C23—H23A	0.9300
			
O1—S1—O3	114.45 (13)	C11—C12—C13	124.8 (2)
O1—S1—O2	112.79 (13)	C11—C12—H12A	117.6
O3—S1—O2	113.43 (12)	C13—C12—H12A	117.6
O1—S1—C24	104.83 (11)	C14—C13—C17	117.1 (2)
O3—S1—C24	105.43 (12)	C14—C13—C12	122.8 (2)
O2—S1—C24	104.70 (11)	C17—C13—C12	120.0 (2)
C15—N1—C16	120.2 (2)	C15—C14—C13	121.0 (2)
C15—N1—C18	119.8 (2)	C15—C14—H14A	119.5
C16—N1—C18	120.0 (2)	C13—C14—H14A	119.5
C10—C1—C2	121.4 (3)	N1—C15—C14	120.1 (2)
C10—C1—H1A	119.3	N1—C15—H15A	119.9
C2—C1—H1A	119.3	C14—C15—H15A	119.9
C3—C2—C1	120.4 (3)	N1—C16—C17	121.2 (2)
C3—C2—H2A	119.8	N1—C16—H16A	119.4
C1—C2—H2A	119.8	C17—C16—H16A	119.4
C2—C3—C4	120.6 (3)	C16—C17—C13	120.2 (2)
C2—C3—H3A	119.7	C16—C17—H17A	119.9
C4—C3—H3A	119.7	C13—C17—H17A	119.9
C9—C4—C5	119.4 (3)	N1—C18—H18A	109.5
C9—C4—C3	120.2 (3)	N1—C18—H18B	109.5
C5—C4—C3	120.4 (3)	H18A—C18—H18B	109.5
C6—C5—C4	120.9 (3)	N1—C18—H18C	109.5
C6—C5—H5A	119.6	H18A—C18—H18C	109.5
C4—C5—H5A	119.6	H18B—C18—H18C	109.5
C5—C6—C7	119.8 (3)	C24—C19—C20	120.3 (2)
C5—C6—H6A	120.1	C24—C19—H19A	119.8
C7—C6—H6A	120.1	C20—C19—H19A	119.8
C8—C7—C6	120.9 (3)	C19—C20—C21	119.1 (3)
C8—C7—H7A	119.5	C19—C20—H20A	120.5
C6—C7—H7A	119.5	C21—C20—H20A	120.5
C7—C8—C9	120.2 (3)	C22—C21—C20	121.3 (2)
C7—C8—H8A	119.9	C22—C21—Cl1	119.7 (2)
C9—C8—H8A	119.9	C20—C21—Cl1	119.0 (2)
C4—C9—C8	118.6 (2)	C21—C22—C23	119.2 (2)
C4—C9—C10	117.9 (2)	C21—C22—H22A	120.4
C8—C9—C10	123.4 (2)	C23—C22—H22A	120.4
C1—C10—C9	119.4 (2)	C22—C23—C24	120.0 (3)
C1—C10—C11	121.0 (3)	C22—C23—H23A	120.0
C9—C10—C11	119.6 (2)	C24—C23—H23A	120.0
C12—C11—C10	124.1 (2)	C19—C24—C23	120.0 (2)
C12—C11—H11A	118.0	C19—C24—S1	120.30 (18)
C10—C11—H11A	118.0	C23—C24—S1	119.6 (2)
			
C10—C1—C2—C3	−0.5 (5)	C17—C13—C14—C15	−0.3 (4)
C1—C2—C3—C4	−1.2 (5)	C12—C13—C14—C15	177.2 (3)
C2—C3—C4—C9	2.4 (4)	C16—N1—C15—C14	2.0 (4)
C2—C3—C4—C5	−175.6 (3)	C18—N1—C15—C14	−176.2 (3)
C9—C4—C5—C6	−0.9 (4)	C13—C14—C15—N1	−1.3 (4)
C3—C4—C5—C6	177.1 (3)	C15—N1—C16—C17	−1.1 (4)
C4—C5—C6—C7	3.1 (5)	C18—N1—C16—C17	177.1 (3)
C5—C6—C7—C8	−2.2 (5)	N1—C16—C17—C13	−0.5 (5)
C6—C7—C8—C9	−0.9 (5)	C14—C13—C17—C16	1.2 (4)
C5—C4—C9—C8	−2.1 (4)	C12—C13—C17—C16	−176.4 (3)
C3—C4—C9—C8	179.8 (3)	C24—C19—C20—C21	−0.3 (4)
C5—C4—C9—C10	176.3 (3)	C19—C20—C21—C22	1.8 (4)
C3—C4—C9—C10	−1.8 (4)	C19—C20—C21—Cl1	−179.12 (19)
C7—C8—C9—C4	3.0 (4)	C20—C21—C22—C23	−1.6 (4)
C7—C8—C9—C10	−175.3 (3)	Cl1—C21—C22—C23	179.36 (18)
C2—C1—C10—C9	1.1 (4)	C21—C22—C23—C24	−0.2 (4)
C2—C1—C10—C11	180.0 (3)	C20—C19—C24—C23	−1.4 (4)
C4—C9—C10—C1	0.1 (4)	C20—C19—C24—S1	176.12 (18)
C8—C9—C10—C1	178.4 (3)	C22—C23—C24—C19	1.7 (4)
C4—C9—C10—C11	−178.8 (3)	C22—C23—C24—S1	−175.90 (18)
C8—C9—C10—C11	−0.5 (4)	O1—S1—C24—C19	142.5 (2)
C1—C10—C11—C12	−33.3 (4)	O3—S1—C24—C19	21.3 (2)
C9—C10—C11—C12	145.6 (3)	O2—S1—C24—C19	−98.6 (2)
C10—C11—C12—C13	179.6 (3)	O1—S1—C24—C23	−40.0 (2)
C11—C12—C13—C14	−11.4 (5)	O3—S1—C24—C23	−161.1 (2)
C11—C12—C13—C17	166.1 (3)	O2—S1—C24—C23	79.0 (2)

### Hydrogen-bond geometry (Å, °)

**Table:**

*D*—H···*A*	*D*—H	H···*A*	*D*···*A*	*D*—H···*A*
C5—H5A···O3	0.93	2.51	3.370 (3)	153
C11—H11A···O1^i^	0.93	2.34	3.267 (3)	178
C14—H14A···O1^i^	0.93	2.38	3.260 (3)	158
C15—H15A···O2^ii^	0.93	2.42	3.285 (3)	155
C17—H17A···O3^iii^	0.93	2.43	3.348 (3)	171
C18—H18A···O2^ii^	0.96	2.45	3.372 (4)	160
C20—H20A···O1^iv^	0.93	2.34	3.226 (4)	159
C22—H22A···O2^v^	0.93	2.52	3.277 (3)	139
C1—H1A···Cg4^iii^	0.93	2.98	3.682 (3)	133
C3—H3A···Cg4^vi^	0.93	2.87	3.651 (3)	142
C6—H6A···Cg3^vii^	0.93	2.82	3.593 (3)	141

Symmetry codes: (i) −*x*+1, −*y*+1, *z*−1/2; (ii) −*x*+3/2, *y*+1/2, *z*−1/2; (iii) *x*+1, *y*, *z*; (iv) *x*, *y*, *z*−1; (v) *x*−1/2, −*y*+1/2, *z*; (vi) *x*+1/2, −*y*+1/2, *z*; (vii) −*x*+1, −*y*+1, *z*+1/2.
